# Vanadyl Sulfate Effects on Systemic Profiles of Metabolic Syndrome in Old Rats with Fructose-Induced Obesity

**DOI:** 10.1155/2018/5257216

**Published:** 2018-12-25

**Authors:** Diego Ortega-Pacheco, María Marcela Jiménez-Pérez, Jeanet Serafín-López, Juan Gabriel Juárez-Rojas, Arturo Ruiz-García, Ursino Pacheco-García

**Affiliations:** ^1^Universidad de la Sierra Sur, Miahuatlán city, State of Oaxaca, Mexico; ^2^Renal PathophysiologyLaboratory, Department of Nephrology, Instituto Nacional de Cardiología “Ignacio Chávez”, Mexico City, Mexico; ^3^Departament of Immunology, Escuela Nacional de Ciencias Biológicas (ENCB), Instituto Politécnico Nacional (IPN), Mexico City, Mexico; ^4^Department of Endocrinology, Instituto Nacional de Cardiología “Ignacio Chávez”, Mexico City, Mexico

## Abstract

**Background:**

Currently, energy obtained from hypercaloric diets has been part of the obesity and type 2 diabetes mellitus (T2DM) epidemics from childhood to old age. Treatment alternatives have been sought from plants, minerals, and trace elements with metabolic effects. Vanadyl sulfate (VS) has been investigated as a hypoglycemic compound in animal and human studies showing effective insulin-mimetic properties. This characteristic encompasses several molecules that have beneficial pleiotropic effects. *The aim* was to determine the antiobesity, hypoglycemic, and hypolipidemic effects of VS on fructose-induced metabolic syndrome in aged rats.

**Material and Methods:**

Five groups of male Wistar rats were made, each with six rats: two groups with normal diet (ND) and three with high-fructose diet (HFD). The first ND group was treated with saline solution (SS), the second with VS; treatment for HFD groups was in the first group with SS, second with VS, and third with metformin. Weight, body mass index (BMI), blood glucose, and lipidic profile were measured; water, food, fructose and energy consumption were also determined. All parameters were compared among groups.

**Results and Discussion:**

Although obese rats treated with VS presented anorexia, oligodipsia, and a marked weight loss in the first two weeks. They recovered food and water intake in the third week with a slow recovery of some weight weeks later. VS normalized blood glucose level and decreased triglyceride and insulin levels in obese rats. These results suggest that vanadyl sulfate shows antiobesity, hypoglycemic, and hypolipidemic properties in old obese rats and could be useful as an alternative, additional, and potent preventive treatment for obesity and T2DM control in elderly obese and poorly controlled diabetic patients.

**Conclusion:**

VS could play an important role in the treatment of metabolic syndrome, contributing to a decrease in obesity and T2DM, through different ways, such as euglycemia, satiety, weight loss, and lipid profile optimization, among others. However, more research is needed to confirm this suggestion.

## 1. Introduction

The prevalence of diabetes mellitus type 2 (T2DM) increased from 1980 to 2014 approximately 4 times and follows this pattern according to the projections of the World Health Organization [1, 2]. It is a serious public health problem with a high health burden taking top positions in the public agenda of both developing countries, such as Mexico, and developed countries [3, 4]. Indeed, the increase is due to aspects such as sedentary lifestyle, high consumption of high-energy diets resulting in obesity, insulin resistance, hyperglycemia, dyslipidemia, hypertension, and systemic inflammation. This brings with it several serious known complications [5]. A risk phase to develop T2DM and cardiovascular and hepatic diseases is the metabolic syndrome (MetS), which is defined as the combination of insulin resistance with three or more of the metabolic abnormalities mentioned above [6].

MetS engages several vital organs and tissues that maintain optimal health when functioning properly. As is known during old age, this function decreases, so when developing morbid processes, the organism degenerates faster, leading to a deplorable quality of life [7]. They especially develop an acute inflammatory profile that promotes greater metabolic abnormalities and cardiovascular complications [8–11]. Frequently, these patients do not respond adequately to conventional pharmacotherapy and their adherence to a diet and exercise programs is nil, so that alternative treatment based on new synthetic drugs, secondary metabolites, or minerals is necessary [12, 13].

Vanadium derivatives such as vanadyl sulfate (VS), sodium orthovanadate, and vanadium complexes with several ligands have shown hypoglycemic effects in animal models and humans [14–16]. In addition, due to their role on insulin signaling and enzymatic process regulation, these compounds are used to treat diabetes, obesity [17], hypertension [18], endothelial dysfunction [19], cancer [20], and mainly the effects in patients with poorly controlled T2DM [21, 22]. VS reduces fasting plasma glucose, glycated hemoglobin (HbA1c), low-density-lipoprotein cholesterol, triglycerides, and body mass index (BMI). These improvements have been shown to be maintained for 2 weeks after the end of administration [23, 24]; compared to orthovanadate, VS shows insulinic properties at very low doses, reducing the presence of adverse effects [16, 25].

In spite of these beneficial effects, vanadium compounds have not been approved for human therapeutic use, because they showed some undesirable collateral effects such as gastrointestinal alteration [14]. However, in older patients with morbid obesity and MetS or patients with poorly controlled T2DM, beneficial effects could be more important than those undesirable effects [18, 22]. Several studies showed that dietary fructose has a direct impact on hepatic lipid metabolism by bypassing the enzyme phosphofructokinase, the regulatory step imposed on glucose. This allows unregulated flow of fructose-derived carbons into lipogenesis, decreasing lipolysis and increasing plasma fasting and postprandial very low-density-lipoprotein triacylglycerols, whole-body lipid oxidation, and lipid droplets, among others [26–29]. In rats, high-fructose diets induce obesity and can be used as metabolic syndrome in an animal model [30–32]. The aim of the present study was to determine the antiobese, hypoglycemic, and hypolipidemic effects of VS on fructose-induced MetS in aged rats.

## 2. Experimental Development

### 2.1. Animals and Diets

All experimental procedures were approved by the Bioethics and Research Committees of the *Instituto Nacional de Cardiología Ignacio Chávez* and were performed in accordance with the Mexican Federal Regulation for Animal Experimentation and Care (NOM-062-ZOO-2001). Male Wistar rats were obtained from the institutional animal facilities.

Male young Wistar rats (6-8 weeks of age) weighing 180–220 g were maintained in their housing conditions under controlled humidity (55%) at 21 ± 1°C temperature with a 12 h : 12 h light : dark cycle.

Control diet was from Harlan Laboratories (2018S Teklad Global 18% protein rodent diet), containing 4.07 kcal/g (18.6% proteins, 44.2% carbohydrates, and 6.2% fat). Rats were initially divided into two groups and treated for 6 months under the following conditions: the normal diet (ND) group with regular chow and drinking water (negative control) and the high-fructose diet (HFD) group was kept with normal chow and 15% fructose (4 kcal/g) in the drinking water.

### 2.2. Administration of Vanadyl Sulfate

On the 28th week, rats of the ND group with similar weights were distributed in two subgroups, and the HFD group was subdivided in three subgroups. These subgroups were followed for the next 4 weeks to confirm no changes in body weight and water and food intake. After that, on the 32nd week, one ND subgroup was treated with isotonic saline solution (100 ml/kg/day) (ND + SS), and the other ND group was treated with vanadyl sulfate (Sigma-Aldrich, USA), 2.72 mg/kg/day (0.015 mmol), which contains 0.750 mg of elemental vanadium, dissolved in SS (ND + VS). On the other hand, one HFD group received saline solution (HFD + SS), the other received metformin (MET) (Sigma-Aldrich, USA) (100 mg/kg/day) (HFD + MET), and the other vanadyl sulfate (2.72 mg/kg/day) (HFD + VS). Metformin and vanadyl sulfate were administered intragastrically.

VS dosage was determined based on previous studies of vanadium [15, 33, 34]. All groups were maintained under the initial diet conditions, and drug treatments were administered daily for 8 weeks. Metformin dosage was determined based on previous studies of metformin treatment [31, 35–37].

### 2.3. Clinical Measurements and Samplings

Body weight (BW) was measured weekly, body length (nasoanal length; mm) monthly, and food and water consumption was measured daily which allows the kilocalorie consumption estimation. Serum samples were obtained at 32 and 40 weeks (before and after drug treatments). An oral glucose tolerance test (OGTT) was performed at week 40, and finally, at the end of the test (week 8 after treatment), animals were anesthetized with pentobarbital; blood samples were obtained by cardiac puncture for biochemical analysis, and liver and retroperitoneal white adipose tissue (WAT) were obtained too. Serum and organs were immediately placed in liquid N_2_ and conserved at −70°C until use for analysis.

Body mass index (BMI) was calculated from the formula BMI = body weight (g)/length^2^ (cm^2^) [32].

Adiposity index was expressed as a percentage of the ratio of WAT (retroperitoneal adipose tissue) weight (g), divided by the total BW (g) at the time of death, multiplied by 100.

### 2.4. Oral Glucose Tolerance Test (OGTT)

At the end of experimentation (week 40), OGTT was performed in 12 h fasted rats. Glucose was administered intraperitoneally at a final dose of 2 g/kg body weight (dissolved in purified saline solution), and glucose plasma levels were measured at 0, 15, 30, 45, 60, 90, 120, 150, and 180 min using an Accutrend Sensor glucometer (Roche).

### 2.5. Biochemical Parameters

Glucose, triglycerides, and total and high-density-lipoprotein cholesterol concentrations were determined in plasma with colorimetric-enzymatic methods (Roche Diagnostics GmbH, Mannheim, Germany) using a Hitachi 902 autoanalyzer (Hitachi Ltd, Tokyo, Japan) using blood samples drawn from the tail vein on weeks 32 and 40 in 12 h fasted animals [38]. Accuracy and precision of lipid measurements are under periodic surveillance by the Centers for Disease Control and Prevention Services (Atlanta, GA, USA). Interassay coefficients of variation were less than 6% for all these assays.

### 2.6. Insulin Quantification

Serum insulin was quantified using a commercial rat ELISA strip purchased from Crystal Chem (USA), as described in the manufacturer's protocol to attain the corresponding profile.

### 2.7. Statistical Analysis

Data were analyzed through SPSS 22 and described as mean ± SEM. Statistical significance was calculated by one-way analysis of variance (ANOVA) (Dunnett's *post hoc* test) to examine the statistical significance among experimental groups *vs.* control. The null hypothesis was rejected when *P* < 0.05 [39].

## 3. Results

### 3.1. HFD-Induced Body Weight Gain and Food Intake Disorder

Over the 28 weeks of evolution, rats fed HFD increases body weight and body mass index as compared to the ND group (*P* < 0.05). These latter rats maintained their weights over the 4 weeks before the VS treatment. Food intake decreased in rats with HFD compared with ND rats, but their water intake was higher (see Supplementary Materials (available here)). This decreased food consumption and increased water consumption were observed from the third or fourth week after initiating HFD, as reported for this model [31, 40].

**Figure 1:**
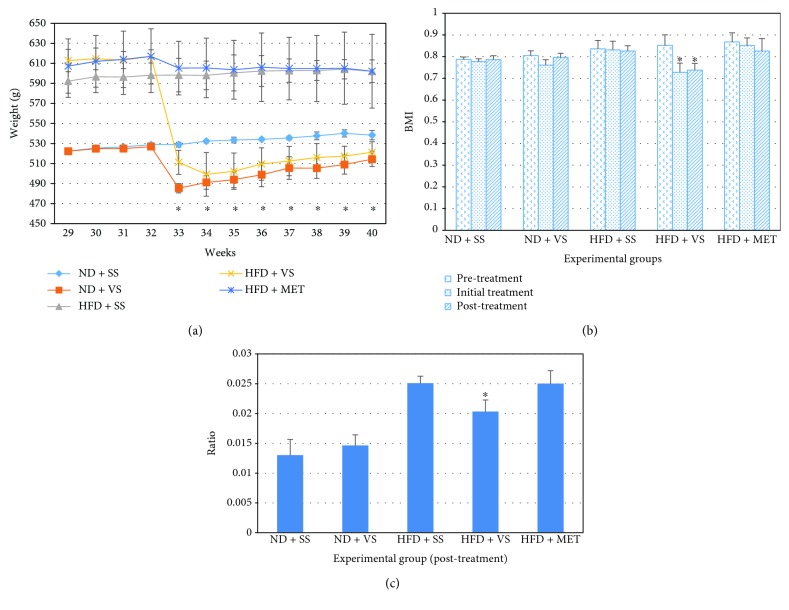
Vanadyl sulfate (VS) effect on body mass of old obese male Wistar rats. Obesity was induced in rats with high-fructose diet (HFD) during 28 weeks, using a set of normal diet (ND) rats as comparative groups; stability in weight was observed during the next 4 weeks. After that, insulin sensitizer treatment (metformin (MET) or VS) was applied from week 32 to week 40, using saline solution (SS) as a control. (a) Body mass evolution after treatment with ND or HFD, and insulin sensitizers (starting week 32). ^∗^ *P* < 0.05 comparing the HFD + VS group versus HFD plus MET or SS. (b) Body mass index (BMI) in animals after 28 weeks on ND or HFD (pretreatment bars), animals with a week of insulin sensitizer treatment (initial treatment bars), and animals with 8 weeks of insulin sensitizer treatment (posttreatment bars). ^∗^ *P* < 0.05 comparing pretreatment versus initial treatment or posttreatment. (c) Abdominal fat tissue/body weight ratio in old obese male Wistar rats after 8 weeks with insulin sensitizer treatment. ^∗^ *P* < 0.05 comparing the HFD + VS group versus HFD plus MET or SS. Data are mean ± SEM (*n* = 6).

Since the beginning of the administration of fructose in drinking water, the rats with a high-fructose diet show an increased consumption of fructose solution and gradually a decrease in the consumption of normal food, and they showed more gain of weight compared with ND rats. Initially, total caloric intake is higher in rats that consume fructose, but after week 20 of the HFD, their total calory consumption begins to decrease (data not shown); it is then lower than that of the normal diet rats (Supplementary Materials). However, these rats do not decrease their body weight and as it is seen at the end, their fat/body weight ratio is higher compared to normal diet rats (Figure 1(c)); this could be due to the fact that due to its chronic obesity status, the rats have less caloric expenditure compared with normal diet rats.

### 3.2. VS Induced Body Weight Decrease in Both Aged Normal and Obese Rats

During the first week of treatment, the obese rats with VS showed a decrease in weight compared to the other experimental groups (*P* < 0.05). In Figure 1(a), it can be seen how the groups treated with VS decrease their weight and the behavior of the weight is similar between the groups under treatment with VS (*P* > 0.05). This difference is maintained until the last week of treatment in the study. However, as the treatment time prolonged, the weight evolution of the VS-treated groups seemed to approach that of the ND + SS group. This aspect is of interest since an optimization of weight is inferred through the use of VS.

The BMI was calculated in each experimental group, before, during, and after the treatment (Figure 1(b)). In fact, at the end of the treatment, it was decreased in the HFD + VS group (*P* < 0.0001); however, in rats of the ND + VS group, there was no significant difference (*P* > 0.05). With respect to the adipose tissue/body weight ratio, the rats under the diet with fructose and treated with VS showed a decreased ratio (*P* = 0.05), which indicates a decrease in fat tissue with respect to body weight.

### 3.3. VS Induced Decreased Food and Fructose-Water Intake in Old Obese Rats

**Figure 2:**
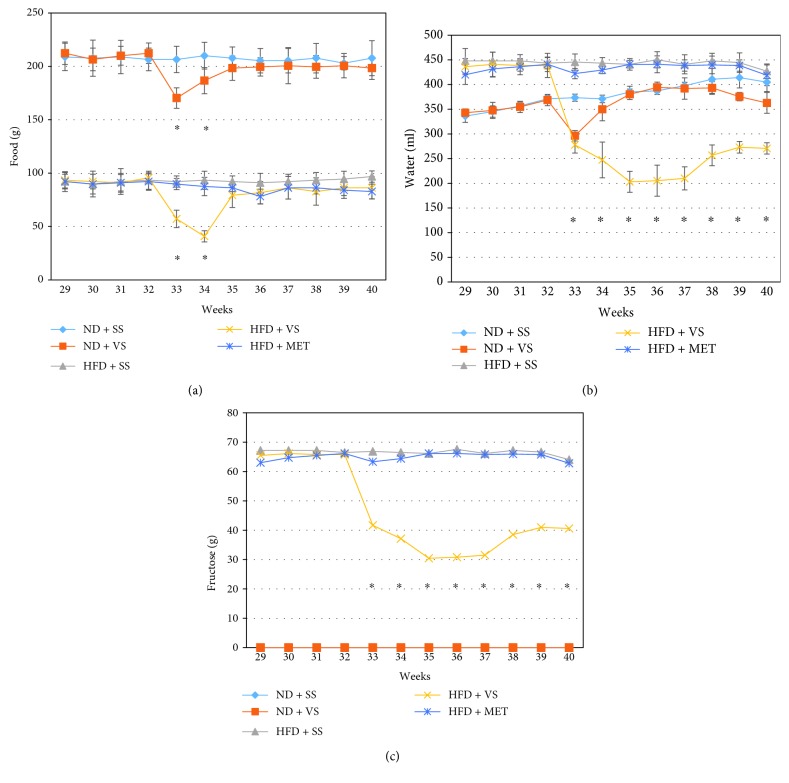
Vanadyl sulfate (VS) effect on food, liquid, and fructose consumption in old obese male Wistar rats. Obesity was induced in rats with high-fructose diet (HFD) during 28 weeks, using a set of normal diet (ND) rats as comparative groups; stability in food and liquid consumption was observed during 4 weeks. After that, insulin sensitizer treatment (metformin (MET) or VS) was applied from week 32 to week 40, using saline solution (SS) as a control. (a) Weekly food intake after treatment with insulin sensitizers (starting week 32) in both lean (ND) and obese (HFD) rats. ^∗^ *P* < 0.05 comparing VS treatment versus MET and/or SS treatment in lean or obese animals. (b) Weekly water drink consumption after treatment with insulin sensitizers. ^∗^ *P* < 0.05 comparing HFD + VS treatment versus HFD + MET or SS treatment. (c) Weekly fructose consumption after treatment with insulin sensitizers. ^∗^ *P* < 0.05 comparing HFD + VS treatment versus HFD + MET or SS treatment. Data are mean ± SEM (*n* = 6).

**Figure 3:**
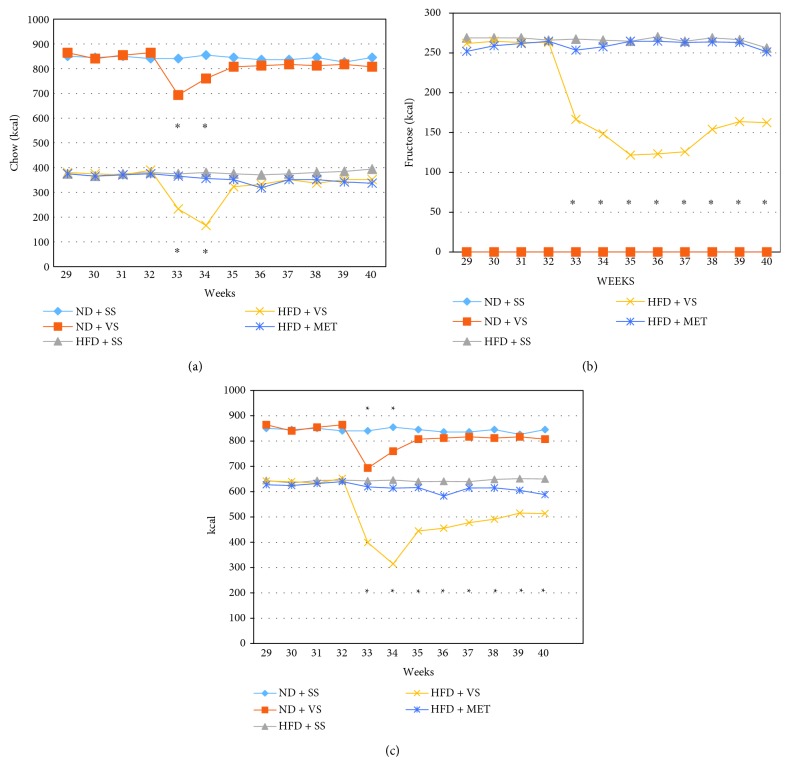
Vanadyl sulfate (VS) effect on calorie intake in old obese male Wistar rats. After obesity induced with high-fructose diet (HFD) during 28 weeks, stability in food and liquid consumption was observed during 4 weeks; then, insulin sensitizer treatment (metformin (MET) or VS) was applied from week 32 to week 40, using saline solution (SS) as a control. Kilocalories were estimated considering the amounts of food and fructose consumption registered per week. (a) Weekly food kilocalorie intake in all experimental groups. ^∗^ *P* < 0.05 comparing VS treatment versus MET and/or SS treatment in both lean and obese animals. (b) Weekly fructose kilocalorie intake after treatment with insulin sensitizers. ^∗^ *P* < 0.05 comparing HFD + VS treatment versus HFD + MET or SS treatment. (c) Weekly total kilocalorie intake in all experimental groups. ^∗^ *P* < 0.05 comparing VS treatment versus MET and/or SS treatment in both lean and obese animals. Data are mean values (*n* = 6).

From the first day of treatment, HFD + VS rats showed decreased food and fructose-water consumption (*P* < 0.0001) (Figures 2(a) and 2(c)) and decreased total calorie consumption, as compared to all other groups (Figure 3). Anorexia and oligodipsia were less remarkable in ND rats receiving VS, although the differences are significant (*P* < 0.0001). ND rats normalized their water intake in the second week after VS administration (*P* = 0.409), and their food intake remained decreased for the 8 weeks of VS treatment. In the third week, the differences between the two VS-treated groups were not significant (Figures 2(b) and 2(c)).

Obese rats treated with VS slowly increased food consumption at the third week of treatment, although not reaching the level before treatment. In the fourth week of treatment, their food consumption was higher than that of the HFD + SS and HFD + MET groups. Nevertheless, these differences were not statistically significant (*P* = 0.966 and *P* = 0.440; respectively). This feeding pattern continued until the eighth week. In the same group, fructose-water consumption decreased in the first week of treatment (*P* < 0.0001), but it increased slowly at the sixth week of treatment, although not reaching the level before treatment (*P* < 0.0001). In the sixth week post-treatment, their water consumption was lesser than that of ND + SS and ND + VS groups, revealing less water intake as compared to the other HFD groups too (*P* < 0.0001) (Figure 2(c)). These results show a possible VS effect on the food consumption regulation in rats.

### 3.4. VS Induced Decreased Calorie Intake in Aged Obese Rats

In general, food intake is decreased in rats with HFD compared to ND rats, while they drank more water than did ND rats. Although drinking water contained fructose at 15%, their total calorie intake was lower in HFD rats than in ND rats because they did not eat much food. At the beginning of treatments, calorie consumption was reduced in rats under VS treatment, as the latter induced anorexia and oligodipsia immediately from the first week (*P* < 0.05); from the third week on, obese rats and obese rats treated with VS recovered their total calorie intake, mainly from food intake. At the sixth week of treatment, these groups recovered the original levels of calories from fructose. ND rats treated with VS remained with less total calorie intake during the 8 weeks of treatment as compared to the nontreated ND rats (Figures 3(a)–3(c)).

### 3.5. VS Induced Decreased Blood Glucose, Insulin Level, and Insulin Resistance in Both Aged Normal and Obese Rats

**Figure 4:**
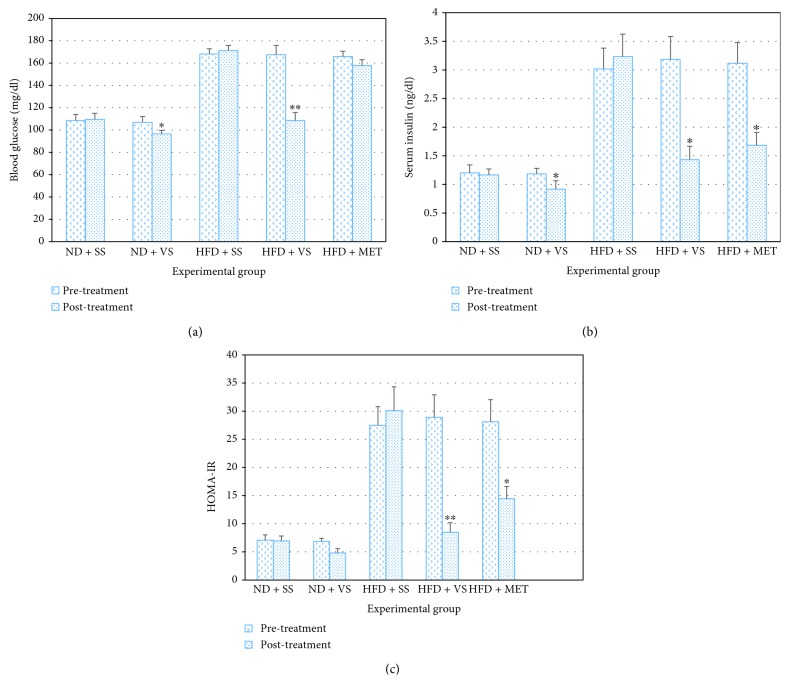
Effect of VS on serum glucose and insulin levels in old obese male Wistar rats. After obesity induced with high-fructose diet (HFD) during 28 weeks, insulin sensitizers treatment (metformin (MET) or VS) was applied from week 32 to week 40, using saline solution (SS) as a control. (a) Postprandial blood glucose levels in rats after 32 weeks on ND or HFD (pretreatment bars) and after 8 weeks of insulin sensitizer treatment (posttreatment bars). ^∗^ *P* < 0.05 comparing ND + VS pre-treatment versus ND + VS post-treatment. ^∗∗^ *P* < 0.05 comparing HFD + VS post-treatment versus HFD plus SS or MET post-treatment. (b) Postprandial blood insulin levels in rats after 32 weeks on ND or HFD (pretreatment bars) and after 8 weeks of insulin sensitizer treatment (post-treatment bars). ^∗^ *P* < 0.05 comparing pre-treatment versus post-treatment. (c) Homeostatic model assessment for insulin resistance (HOMA-IR) in rats after 32 weeks on ND or HFD (pretreatment bars) and after 8 weeks of insulin sensitizer treatment (posttreatment bars). ^∗^ *P* < 0.05 HFD + MET pre-treatment versus HFD + SS post-treatment. ^∗∗^ *P* < 0.05 HFD + VS post-treatment versus HFD + VS pre-treatment HFD + SS or HFD + MET post-treatment. Data are mean ± SEM (*n* = 6).

Rats fed with HFD showed moderate increase of blood glucose, compared to the respective control group (*P* < 0.0001), resulting in a compensatory hyperinsulinemia and insulin resistance. Nevertheless, treatment with VS maintained for 8 weeks induced an important blood glucose level decrease in the ND + VS (*P* = 0.002) and HFD + VS (*P* < 0.0001) groups. VS decreased the levels of insulin in the HFD + VS compared to HFD + SS group (*P* = 0.0001). Values showed that VS exerted an important effect on improving the hyperglycemia and insulin resistance in aged obese rats with a chronic high-fructose diet without negatively affecting these levels in the ND group (Figure 4(a)).

When comparing the HFD + MET group to the HFD + VS group, the latter group showed a greater decrease in blood glucose (*P* < 0.0001) and HOMA-IR index (*P* = 0.004). Blood insulin in the HFD + VS group was lower than in the HFD + MET group, but not significantly (*P* = 0.39). This suggests that VS could have a potent hypoglycemic effect than metformin, at least at the dosages used in these experiments (Figures 4(b) and 4(c)).

### 3.6. VS Effect on Blood Triglycerides and Total, HDL, and LDL Cholesterol in Both Old Normal and Obese Rats

**Figure 5:**
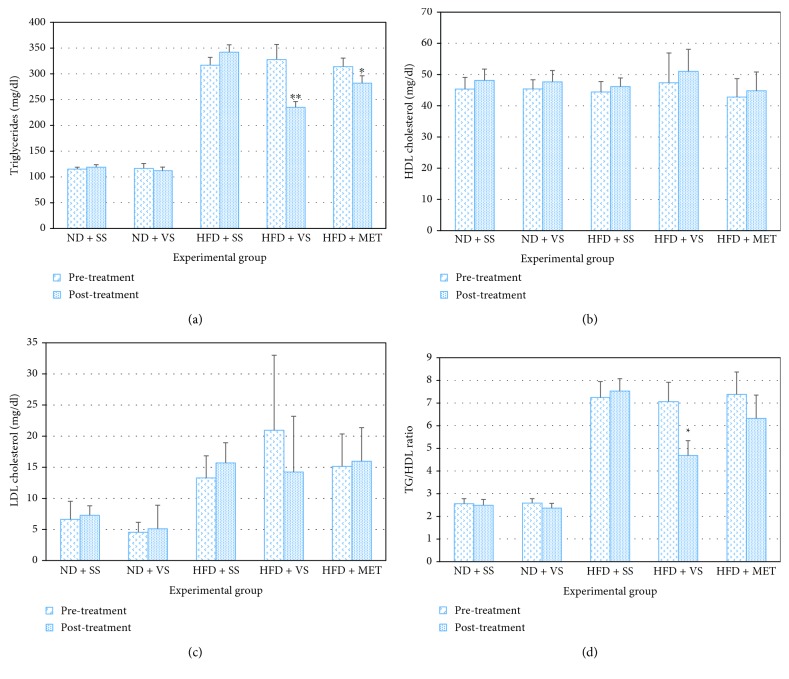
Effect of VS on lipid plasma levels in old obese male Wistar rats. After obesity induced with high-fructose diet (HFD) during 28 weeks, insulin sensitizer treatment (metformin (MET) or VS) was applied from week 32 to week 40, using saline solution (SS) as a control. (a) Triglyceride fasting plasma levels in rats after 32 weeks on ND or HFD (pretreatment bars) and after 8 weeks of insulin sensitizer treatment (posttreatment bars). ^∗^ *P* < 0.05 HFD + SS post-treatment. ^∗∗^ *P* < 0.05 comparing HFD + VS post-treatment versus HFD + SS or HFD + MET post-treatment. (b, c) HDL and LDL cholesterol plasma levels in rats after 32 weeks on ND or HFD (pretreatment bars) and after 8 weeks of insulin sensitizer treatment (posttreatment bars) (nonsignificant differences were found). (d) Triglyceride/HDL ratio in rats after 32 weeks on ND or HFD (pretreatment bars) and after 8 weeks of insulin sensitizer treatment (posttreatment bars). ^∗^ *P* < 0.05 comparing HFD + VS post-treatment versus HFD + SS or HFD + MET post-treatment. Data are mean ± SEM (*n* = 6).

Rats fed with HFD showed a large increase in triglycerides and cholesterol (*P* < 0.05) compared to the respective control group. Treatment with VS maintained for 8 weeks induced a large decrease in triglyceride levels in obese rats compared to control groups (*P* < 0.0001). Triglyceride reduction in ND + VS was not significant (*P* = 0.48). Although VS and metformin increased the levels of HDL cholesterol in this experiment, differences among groups were not significant (*P* = 0.272). Neither did LDL and total cholesterol show significant differences (*P* > 0.05). Metformin induced a slight decrease in these parameters in obese rats. In the ND group, VS did not significantly affect blood triglyceride and cholesterol levels (Figures 5(a)–5(d)).

### 3.7. VS Administration Effect on the Oral Glucose Tolerance Test

**Figure 6:**
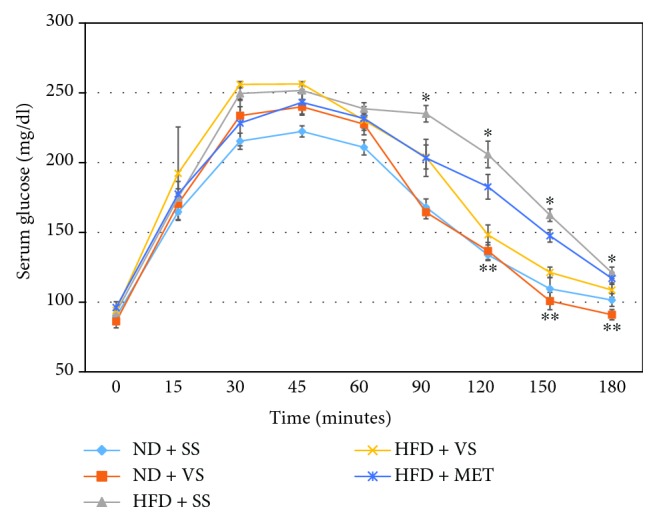
Effect of VS on oral glucose tolerance test in old obese male Wistar rats. Obesity was induced in rats with high-fructose diet (HFD) during 28 weeks, using a set of normal diet (ND) rats as comparative groups; stability in weight was observed during the next 4 weeks. After that, insulin sensitizer treatment (metformin (MET) or VS) was applied from week 32 to week 40, using saline solution (SS) as a control. ^∗^ *P* < 0.05 comparing HFD + SS versus ND + SS. ^∗∗^ *P* < 0.05 comparing HFD + VS versus HFD + SS or HFD + MET. Data are mean ± SEM (*n* = 6).

In this experiment, the OGTT curve was more extended than commonly reported for rats from younger animals or in short-term induced metabolic syndrome (Figure 6). Treatment with VS during 8 weeks improved OGTT in both ND and HFD groups, compared to the control group, and the improvement was better than that induced by metformin in HFD rats. Groups of obese rats represent the highest curves until the 45th minute where the curves begin to fall. Each treatment with VS is compared with its controls. The HFD + VS group represented the highest curve. At the end (180 minutes), the value of 108.6 mg/dl was reached, which is a lower value than the control group for obese rats (*P* < 0.05). Although it represents a borderline value of glycemia, it has a more potent therapeutic effect than metformin, as has been shown in several studies. The ND + VS group represented the curve that was most suitable and was lower than the rats with a normal diet (*P* = 0.05).

## 4. Discussion

Most studies on metabolic syndrome in animal models are made for short periods in young individuals. Humans develop T2DM and other metabolic syndrome complications after many years of bad lifestyles, such as high caloric diets and sedentary life. On the other hand, adults or aged people are the ones to present the worst MetS-derived complications and for whom the treatment is more difficult. For these reasons, we approached the study of vanadyl sulfate treatment on a long-term metabolic syndrome, using a similar animal model.

Our interest was to study the possible beneficial effects of vanadyl sulfate on the treatment of metabolic syndrome complications in adult human patients, who develop obesity, morbid obesity, and T2DM. Vanadyl sulfate effects on biochemical, anthropometrical, and food intake parameters were measured in rats that developed obesity through a diet high in fructose and compared to normal diet rats.

Fructose is present in sweeteners, like sucrose and high-fructose corn syrup (HFCS), in most industrialized food products; high sugar consumption has severe consequences for health, such as diabetes, hypertension, obesity development, uric acid accumulation, and a proinflammatory effect [26, 41]. In fact, several physiological mechanisms are related to a high-fructose diet such as insulin signaling and peripheral and central satiety [29].

High-fructose diet (HFD) is a model used in laboratory animals to induce body weight increase, obesity, and metabolic syndrome; rats fed HFD show increased body weight and body mass index. Food intake is decreased in rats given HFD compared with ND rats, but the intake of fructose-water is increased, resulting in increased total calorie consumption. This decreased food and increased water consumption was observed from the third or fourth week of HFD in our experiment similarly to other reports for this model in short-term experiments. However, in our long-term obesity trial, old rats with HFD showed a diminished total calorie consumption without loss of body weight. This decrement is shown after the 20th week of HFD (Supplementary Materials).

Vanadium is an omnipresent and essential micronutrient in the human diet through different inorganic compounds, such as VS, sodium metavanadate, sodium orthovanadate, and vanadium pentoxide [14, 42]. Although vanadium is toxic at high doses, its absorption, which is mediated by the duodenum, is poor [14, 23]. VS has pleiotropic organic effects like regenerative, antihypertensive, immune modulator, antitumor, and hypoglycemic [14, 20, 22, 23, 43].

In this study, VS was orally administered, dissolved in saline solution, because some authors have reported that rats drink less water when vanadium compounds are administrated in drinking water, arguing that this is due to an unpleasant flavor. Our results show that VS had an anorexic effect, diminishing body weight, BMI, food, water, and fructose intake in obese and nonobese rats.

VS was applied at a dose of 2.72 mg/kg/day; this dose was calculated to provide 0.750 mg/kg/day of the vanadium element. Oral administration allows us to control the amount of VS administered, because when it is administered in drinking water, rats decrease the consumption of water in a variable manner, which has been attributed to the bad taste. However, in this experiment we observed that both the animals that receive water alone (unflavored) and those that receive sweetened water with fructose decrease their water consumption after the administration of VS.

On the other hand, when we apply this same dose of VS intraperitoneally or subcutaneously, VS shows the same effects, but the rats show constant anorexia and oligodipsia with chronic cachexia and die in the second or third week of administration, even if the treatment is withdrawn (data not shown). In contrast, rats that received oral VS at this dose show a cachexic state at the beginning, but they recover from this aspect; some rats have mild diarrhea and all show greenish-colored stool throughout the treatment, indicating that part of the VS is not absorbed and is eliminated by fecal route. The recovery from its bad general state could be attributed to the fact that rats diminish their capacity to uptake VS from the gastrointestinal tissue. They improve their capacity of elimination of the VS by fecal way and/or that they adapt to eliminate or transform VS mainly in the liver. These adaptation mechanisms cannot develop them when VS is administered subcutaneously or intraperitoneally.

During treatment, BMI values were similar in both groups until the last week. In this time, the BMI of the HFD and ND with VS groups stayed within error of the values of the normal group without treatment. BMI and weight reduction started together with food, water, and fructose intake decrement. Nevertheless, food intake increased in the third treatment week until it was equal to the respective control group. Although food intake is normalized in a few weeks, BMI, water, and fructose are not. Water intake in obese rats was maintained among all groups until the last week, but in ND-treated rats, consumption was recovered. This effect has been seen in other studies [18]. Despite these results, fructose consumption remained diminished in obese rats treated with VS for the first week until the eighth week. These characteristics suggest that VS could be a fructose intake regulator or a sweet beverage regulator independently from the food intake. If this aspect is applicable to humans, it could be used to improve current consumption of beverages with high fructose without affecting the food consumption, thereby improving the anthropometric profile as a preventive measure and contributing to public health.

Fructose decreases central and peripheral satiety through inhibition of neuropeptide YY (PYY), ghrelin, and proopiomelanocortin (POMC) mRNA or by an increase in circulating leptin levels [29, 44]. Additionally, leptin resistance is observed in animal models with high lipids in their diet and it is related to inflammation that decrease TNF-*α* and its toxicity [45]. Nevertheless, hormones like insulin, glucocorticoids, and estrogens are positive leptin regulators [44]; therefore, the insulin-mimetic effect of VS can modify these pathways or molecules, by incrementing satiety for fructose independently from food intake.

Several studies have shown the insulin-mimetic effects of VS, and its hypoglycemic effect is the most investigated currently [18, 21, 23, 46]. In fact, our results are similar, old obese rats treated with VS-diminished glucose and insulin blood levels compared to respective control groups. In fat rats with VS, glucose decrease was more efficient than metformin treatment (*P* > 0.0001), whereas the insulin level decrement was similar in both treatments (*P* = 0.399). ND rats with VS treatment showed a significant blood glucose decrement (*P* = 0.002). Goldfine et al. [21] exhibited the VS effect in T2DM patients; they concluded that VS acts on human skeletal muscle in the early insulin signaling steps. These steps are on basal insulin receptor, substrate tyrosine phosphorylation, and PI-3-kinase activation [21]. In fact, this molecular cascade has action on other enzymes, such as PI3K-PK*β*/Akt-mTOR, NF-*κβ*, and MEK1/2-ERK activation. These messengers are related to GLUT transporter insertion into the plasma membrane, which results in increased glucose transport into the cells and subsequently decreased blood glucose and insulin [21, 23, 47, 48]. Gluconeogenesis and hepatic glucose output are decreased in treatment with vanadium; consequently, this compound could diminish the loss of muscle protein that occurs in T2DM patients [16].

Studies in mice treated with vanadium compounds revealed an antiobesity effect, adiponectin segregation, increment of insulin product, and adipogenic effect [42]. These mechanisms are related to adiponectin, leptin, and insulin hormones and their effects on satiety and body weight [29, 30, 44, 49]. Insulin production increases adiponectin synthesis through adipocytes, improving satiety, which probably influenced the BMI of obese rats in this study, as shown by Maachi et al. [50].

On the other hand, a study in the mouse preadipocyte fibroblast cell line 3T3-L1 showed an adipocyte differentiation inhibitory effect of vanadium dissolved in water through PPAR*γ* and C/EBP*α* gene downregulation [17]. PPAR*γ*, as a peroxisome proliferator, is a nuclear receptor implicated in fat accumulation in adipocytes, weight increment, and leptin negative regulation. It is thiazolidinedione's therapeutic target with secondary effects [44, 51, 52].

It has been evidenced that VS treatment of T2DM patients reduces serum cholesterol, triglycerides, and glucose [21, 25, 46]. In our study, total cholesterol and triglycerides decreased in treated aged rats but differences in other parameters, such as HDL and LDL, were not significant (*P* > 0.05). It should not be discarded that this lack of significance could be due to the fact that results of the present study show a high standard deviation in obese rats treated with vanadyl sulfate (HFD + VS group). Moreover, it has been previously reported that HDL decreases in T2DM patients treated with VS [21]. Although results of the present study show a slightly increased in HDL (*P* = nonsignificant), triglyceride/HDL and fat tissue/body weight ratios were the lowest in VS-treated obese aged rats (*P* = 0.003), suggesting a fat tissue loss and HDL increment. In fact, in our analysis an elevated triglyceride/HDL ratio was correlated with insulin resistance observed in HFD aged rats. These effects can be explained because VS can stimulate other nuclear receptors, like PPAR*γ*, involved in fatty acid mitochondrial oxidation, energy consumption, thermogenesis, HDL increment, and triglyceride decrease as reported by Bermúdez et al. [51].

Obesity is a chronic inflammatory state with different factors involved in its development and many consequences, like T2DM. According to our results, VS can act like a potent preventive dietary compound that improves insulin resistance, hyperglycemia, OGTT, high cholesterol, and high triglyceride levels. Although this study was made in an animal model, currently beneficial properties of VS have been demonstrated in humans by decreasing the same parameters as in this investigation without affecting hepatic enzymes [21]. People consume diets with a high-fructose or -sucrose content; these sugars are elevated in industrialized sweetened beverages and foods with unclear health regulation [53]. Consequently, the entire population is immersed in an obesogenic environment that has several consequences, one of them the metabolic syndrome [41, 54]. Thus, the parameters evaluated show a possible alternative treatment and a preventive dietary compound. It is necessary to investigate the role of VS on fructose as well as on water and food decreased consumption.

## 5. Conclusions

According to our results, VS has an antiobesity effect in aged obese rats. VS induces decreased fructose consumption and oligodipsia, decreasing the total calorie intake inducing the immediate loss of weight in these aged obese rats. VS induces decreases in levels of blood glucose, triglycerides, cholesterol, and triglyceride/HDL ratio and improves insulin sensitivity and oral glucose tolerance test results at 8 weeks of treatment in rats with fructose-induced chronic obesity.

VS can be a valuable therapeutic agent in preventing insulin resistance, as well as the development and progression of obesity and metabolic syndrome complications in aged patients with obesity or T2DM. However, research on undesirable side effects in long-term trials is necessary. With this experiment, we obtained serum and organ samples that permit us to study beneficial or undesirable effects on other metabolic parameters such as inflammatory state, oxidative stress, and organ damage, results that will allow us to evaluate the convenience of vanadyl sulfate administration in human patients.

## Data Availability

The data used to support the findings of this study are available from the corresponding author upon request. Information about the evolution of old rats from week 0 to 25 is available in supplementary data.

## Supplementary Materials

Supplementary Materials This file contains the data obtained during 25 weeks of evolution of the metabolic syndrome in Wistar rats used to be treated with vanadyl sulfate (VS). From the first week, the rats with normal diet (ND) received normal food and pure water as a drink, and the rats with high-fructose diet (HFD) received normal food and water with fructose at 15% as drinking water. All rats had ad libitum access to food and drinking water. At week zero, all the young rats (200-240 g) received pure water as a drink. Body weight, food consumption, drinking water consumption, fructose consumption, and calculation of total kilocalories and from each source are indicated weekly. As indicated in the article, vanadyl sulfate effects on systemic profiles of metabolic syndrome in old rats with fructose-induced obesity these rats were reclassified according to their weight at week 28 and treated with VS from week 32 onwards.
